# Androgen Regulation of 5α-Reductase Isoenzymes in Prostate Cancer: Implications for Prostate Cancer Prevention

**DOI:** 10.1371/journal.pone.0028840

**Published:** 2011-12-14

**Authors:** Jin Li, Zhiyong Ding, Zhengxin Wang, Jing-Fang Lu, Sankar N. Maity, Nora M. Navone, Christopher J. Logothetis, Gordon B. Mills, Jeri Kim

**Affiliations:** 1 Department of Systems Biology, The University of Texas MD Anderson Cancer Center, Houston, Texas, United States of America; 2 Department of Cancer Biology, The University of Texas MD Anderson Cancer Center, Houston, Texas, United States of America; 3 Department of Genitourinary Medical Oncology, The University of Texas MD Anderson Cancer Center, Houston, Texas, United States of America; University of Kentucky College of Medicine, United States of America

## Abstract

The enzyme 5α-reductase, which converts testosterone to dihydrotestosterone (DHT), performs key functions in the androgen receptor (AR) signaling pathway. The three isoenzymes of 5α-reductase identified to date are encoded by different genes: *SRD5A1*, *SRD5A2*, and *SRD5A3*. In this study, we investigated mechanisms underlying androgen regulation of 5α-reductase isoenzyme expression in human prostate cells. We found that androgen regulates the mRNA level of 5α-reductase isoenzymes in a cell type–specific manner, that such regulation occurs at the transcriptional level, and that AR is necessary for this regulation. In addition, our results suggest that AR is recruited to a negative androgen response element (nARE) on the promoter of *SRD5A3 in vivo* and directly binds to the nARE *in vitro*. The different expression levels of 5α-reductase isoenzymes may confer response or resistance to 5α-reductase inhibitors and thus may have importance in prostate cancer prevention.

## Introduction

The multiyear, multistep process of prostate carcinogenesis and its long latency period make prostate cancer ideal for chemoprevention [1]. The androgen receptor (AR) signaling pathway, which is essential for prostate development and normal function, is also central to prostate cancer's pathogenesis and progression [2], [3]. The key enzyme in AR signaling, 5α-reductase, converts testosterone to the more potent androgen dihydrotestosterone (DHT) [4]. Although testosterone can bind to and activate the AR, DHT binds to it with a dissociation rate three times slower than that of testosterone [5], [6].

Three isoenzymes of 5α-reductase, which are encoded by different genes (*SRD5A1*, *SRD5A2*, and *SRD5A3*), have been identified. Immunohistochemical and polymerase chain reaction (PCR) analyses of human prostate tissues suggest that SRD5A1 and SRD5A2 levels change with prostate cancer development and progression [7], [8], [9]. *In vitro* studies have confirmed the 5α-reductase activity of the more-recently identified SRD5A3 [10], which was overexpressed in hormone-refractory prostate cancer tissues [10], [11]. Knockdown of *SRD5A3* expression also reduced the growth and viability of prostate cancer cells [10]. By using a monoclonal antibody, Godoy et al. further showed increased level of SRD5A3 protein in the prostate cancer compared to benign prostate tissues [12]. These findings suggested that SRD5A3 may contribute to prostate cancer progression. It has also been recently reported that SRD5A3 may play an important role in protein glycosylation [13]. Mutations of *SRD5A3* result in congenital disorders [13], [14] and Kahrizi syndrome [15].

Two 5α-reductase inhibitors have been tested clinically. Finasteride specifically inhibits SRD5A2 activity [16], and dutasteride inhibits that of both SRD5A1 and SRD5A2 [17]. The Prostate Cancer Prevention Trial (PCPT) yielded encouraging results: finasteride reduced the overall incidence of prostate cancer by 25%, although potential effects of high-grade tumors were concerning [18]. Similarly, the Reduction by Dutasteride of Prostate Cancer Events (REDUCE) trial showed that dutasteride reduced the incidence of prostate cancer by 23% among men at high risk and revealed no statistically significant increase of high-grade tumor in dutasteride-treated men [19], [20].

Three factors may confer response or resistance to 5α-reductase inhibitors. First, response or resistance may result from the presence of different isoenzymes [21]. Second, differences in sensitivity may be conferred by *SRD5A2* genotypic variants [22]; Makridakis et al. [23] showed *in vitro* that *SRD5A2* variants have different affinities for finasteride. Third, different expression levels of the 5α-reductase isoenzymes could contribute to both sensitivity and resistance. Unlike androgen ablation, which decreases prostatic testosterone and DHT, inhibition of 5α-reductase activity decreases DHT but increases testosterone [24], [25], [26]. Since 5α-reductase inhibitors change the testosterone-to-DHT ratio, and given the critical role of 5α-reductase in AR signaling, the different 5α-reductase expression levels may provide clues about response and resistance to 5α-reductase inhibitors in prostate cancer prevention.

Androgens can affect the expression of *SRD5A1* and *SRD5A2* in different tissues and cell types. In the rat ventral prostate, positive regulation of *SRD5A2* by androgen has been reported [27], and in the rat testis, negative regulation of *SRD5A1* [28]. Androgen ablation led to decreased immunostaining of 5α-reductase [29]. *SRD5A1* and *SRD5A2* are also regulated by testosterone and DHT in T and B lymphoid cells [30] and in rat liver and brain [31], [32], [33], [34]. However, how 5α-reductase expression is regulated in human prostate cells has not been extensively investigated.

Our primary purpose of this study was thus to evaluate androgen regulation of the 5α-reductase isoenzymes in human prostate cells. We further investigated whether the regulatory effects of androgens on the 5α-reductases are mediated by AR and whether a direct interaction exists between the *cis*-regulatory elements of 5α-reductase isoenzymes and AR.

Our data demonstrated cell type–specific androgen regulation of the isoenzymes that is mediated by AR. To our knowledge, this is the first demonstration that AR can directly bind to the negative androgen response element (nARE) of the *SRD5A3* promoter in LNCaP prostate cancer cells. Our findings may have clinical implications for identifying men whose disease may benefit from 5α-reductase inhibitors.

## Materials and Methods

### Cell lines and cultures

PWR-1E, LNCaP, and VCaP cells were obtained from the American Type Culture Collection (ATCC, Manassas, VA); BPH-1-GFP, BPH-1-AR, and C4-2B4 cells were a gift from Dr. Sue-Hwa Lin (The University of Texas MD Anderson Cancer Center, Houston, TX); and LAPC-4 cells were kindly provided by Dr. Robert Reiter (University of California, Los Angeles, CA).

PWR-1E cells were maintained in serum-free keratinocyte medium (Invitrogen, Life Technologies Corp., Carlsbad, CA) supplemented with 50 µg/mL bovine pituitary extract, 5% l-glutamine, and 5 ng/mL epidermal growth factor. LNCaP, C4-2B4, BPH-1-GFP, and BPH-1-AR cells were maintained in RPMI-1640 medium with 10% fetal bovine serum (FBS) and 1% penicillin and streptomycin (P/S). LAPC-4 cells were maintained in Iscove's modified Dulbecco's medium (Invitrogen) supplemented with 5% FBS and 1% P/S. VCaP cells were maintained in Dulbecco's Modified Eagle's Medium supplemented with 10% FBS and 1% P/S. All cultures were maintained at 37°C in humidified air with 5% CO_2_. Cell lines were validated at MD Anderson's Characterized Cell Line Core by STR DNA fingerprinting using the AmpFℓSTR Identifiler kit (Applied Biosystems, Life Technologies Corp., Carlsbad, CA). The STR profiles were compared to the known ATCC fingerprints, to the Cell Line Integrated Molecular Authentication Database version 0.1.200808 (http://bioinformatics.istge.it/clima/) [35], and to MD Anderson's fingerprint database. The STR profiles of PWR-1E, LNCaP, C4-2B4, and VCaP cells matched known DNA fingerprints; those of BPH-1-AR and LAPC-4 cells were unique.

### Quantitative reverse-transcription PCR (qRT-PCR)

Total RNA was extracted from each cell line by using an RNeasy Plus mini kit (Qiagen Inc., Valencia, CA) according to the manufacturer's protocol. qRT-PCR was performed by using a TaqMan One-Step RT-PCR kit (Applied Biosystems, Life Technologies Corp.), according to the manufacturer's instructions. Briefly, the qRT-PCR setting for each reaction was 48°C for 30 minutes, 95°C for 10 minutes, and 42 cycles of 95°C for 15 seconds and 60°C for 1 minute. Human β-actin was used as the endogenous control in each reaction. Primer and probes for *SRD5A1*, *SRD5A2*, and *SRD5A3* genes were also from Applied Biosystems.

### Androgen treatment

Testosterone and DHT were purchased from Sigma-Aldrich (St. Louis, MO). R1881, a synthetic androgen, was kindly provided by Dr. Sue-Hwa Lin. Cells were seeded in 12-well plates with their regular growth medium. After serum starvation overnight, cells were exposed to ethanol (control) or to 1 nM, 10 nM, or 100 nM androgen. After 24-hour and 48-hour incubation, cells were harvested and RNA extracted for qRT-PCR analysis.

### Actinomycin D treatment

BPH-1-AR, LNCaP, and PWR-1E cells were treated with dimethyl sulfoxide (DMSO; control) or 1 µg/mL or 5 µg/mL of actinomycin D for 30 minutes, followed by ethanol (control) or 10 nM DHT. After 24 hours' incubation, cells were harvested and total RNA extracted.

### Transfection with small interfering RNA (siRNA)

To knock down AR expression, we seeded LNCaP cells in 12-well plates, serum starved them overnight, and then transfected them with 20 nM AR siRNA or control siRNA by using DharmaFECT 1 (Dhamarcon, Inc., Thermo Fisher Scientific, Lafayette, CO). After incubation overnight, cells were exposed to ethanol (control) or 2 nM DHT. The AR siRNA and control siRNA were obtained from Dhamarcon.

### Western blot analysis

After 24 hours of incubation with siRNA, the cells were harvested and centrifuged at 5,000 rpm for 5 minutes. Cell pellets were resuspended in RIPA buffer (Boston Bioproducts, Inc., Ashland, MA) with protease inhibitor (Roche, Mannheim, Germany), incubated for 20 minutes with occasional vortex mixing, and then centrifuged at 14,000 rpm for 10 minutes. The supernatant was decanted and saved for Western blotting. The whole protein-extraction procedure was performed at 4°C, and the protein concentration was measured by using the BCA assay (Thermo Fisher Scientific, Inc., Waltham, MA). The supernatant was boiled for 5 minutes, loaded onto polyacrylamide gel, run, and transferred to a PVDF membrane, which was then blocked in TBST (TBS with 0.2% Tween 20) + 5% milk for 1 hour before being probed with anti-AR antibody (Dako North America, Inc., Carpinteria, CA) in blocking buffer overnight at 4°C, followed by incubation at room temperature for 1 hour with secondary horseradish peroxidase–conjugated anti-mouse antibody. Detection was performed by using an electrochemiluminescence kit (Amersham, GE Healthcare Bio-Sciences Corp., Piscataway, NJ).

### Luciferase assay

The *SRD5A3* promoter (−1027/+155 bp) was cloned into the pGL2 basic vector (Promega Corp., Madison, WI) at the XhoI and MluI sites, as were further deletion constructs. LNCaP cells were transfected with these constructs by using Fugene 6 reagent (Roche) or Lipofectamine 2000 (Invitrogen).

To test the AR-dependent repression ability of SRD5A3, we also inserted its promoter (−191/−72 bp) into the pGL3–4ARE–E4–luc construct at the PstI and XhoI sites. LNCaP cells were transfected with it and then grown in the absence and presence of DHT for 24 hours.

A dual-luciferase assay was conducted according to Promega's protocol. The luciferase ratio was derived by dividing the luciferase activity by the *Renilla* activity.

### Mutagenesis

Mutations were made in the nARE region of the *SRD5A3* promoter by using a QuickChange II XL site-directed mutagenesis kit (Strategene, Agilent Technologies, Inc., Santa Clara, CA) according to the manufacturer's instructions. The sequence CTGTTTTGCGTCT was mutated to ATTTTTTTATTAT in the context of the pGL3–4ARE–E4–luc construct.

### Electrophoretic mobility-shift assay (EMSA)

AR's binding to double-stranded oligos was assessed in LNCaP cell nuclear extracts by performing EMSA with a LightShift kit (all kits for EMSA were from Thermo Scientific) according to the manufacturer's instructions. Nuclear protein extracts were isolated from LNCaP cells, and protein concentrations were determined by using a BCA protein assay kit. Oligos were designed to cover the −191/−91 region on the *SRD5A3* promoter and labeled by using a biotin 3′-end DNA labeling kit according to the manufacturer's instructions. AR binding to double-stranded oligos was assessed in LNCaP cell nuclear extracts by EMSA using a LightShift kit according to the manufacturer's instructions. Unlabeled oligos were used in EMSA as one of the controls. Biotin-labeled DNA was detected on the nylon membrane by using a chemiluminescent nucleic acid detection module kit.

### Chromatin immunoprecipitation (ChIP) assay

LNCaP cells were cultured in both the presence and absence of 100 nM DHT, and ChIP was performed by using a kit from Cell Signaling Technology, Inc. (Danvers, MA), according to their protocol. Briefly, cells were treated with 37% formaldehyde to cross-link proteins to DNA. Cross-linked chromatin was digested by micrococcal nuclease to a length of 150–900 bp, and the nuclear membranes were broken by sonication. AR-specific antibodies (Dako) were used to precipitate the short chromatin fragments, which were then washed and eluted. The protein–DNA cross-links were reversed and DNA further purified by using spin columns in each PCR reaction. The forward primer for the nARE was CTGTTTTGCGTCTTTGCTTCTG, and the reverse primer was GAGGTCCTTGGTCCTGGTC, which amplify a 120-bp product.

### Xenograft models

RNA from the MDA PCa 183, MDA PCa 144, MDA PCa 146, and MDA PCa 155 xenograft models was kindly provided by Dr. Sankar N. Maity and Dr. Nora M. Navone. The MDA PCa 183 xenograft was derived from androgen-dependent prostate carcinoma, whereas the others were derived from AR-negative castrate-resistant prostate carcinomas with small-cell prostate carcinoma (SCPC) morphology.

Quantification of the relative mRNA level of xenograft *SRD5A3* and prostate-specific antigen (*PSA*) was done by using qRT-PCR with SYBR Green (Applied Biosystems, Inc.). qRT-PCR was performed as described in [36]. The primer sequences used were as follows: SRD5A3: forward, 5′-TCCAAGCTGGCTTCATGGTT-3′ located on exon 2 and reverse, 5′-CACTCGAAGAGTCTTCGTAA-3′ located on exon 3; PSA: forward, 5′-GAGAGCTGTGTCACCATGTG-3′ located on exon 1 and reverse, 5′-CACAATCCGAGACAGGATGA-3′ located on exon 2.

### Statistical analysis

Data are presented as means ± SD. Two-sided *t* tests were conducted for comparing means between androgen-treated samples and controls. Significance was set at a p value of 0.05.

## Results

### All three 5α-reductase isoenzymes are present in the tested human prostate cell lines, with varying expression patterns

**Figure 1:**
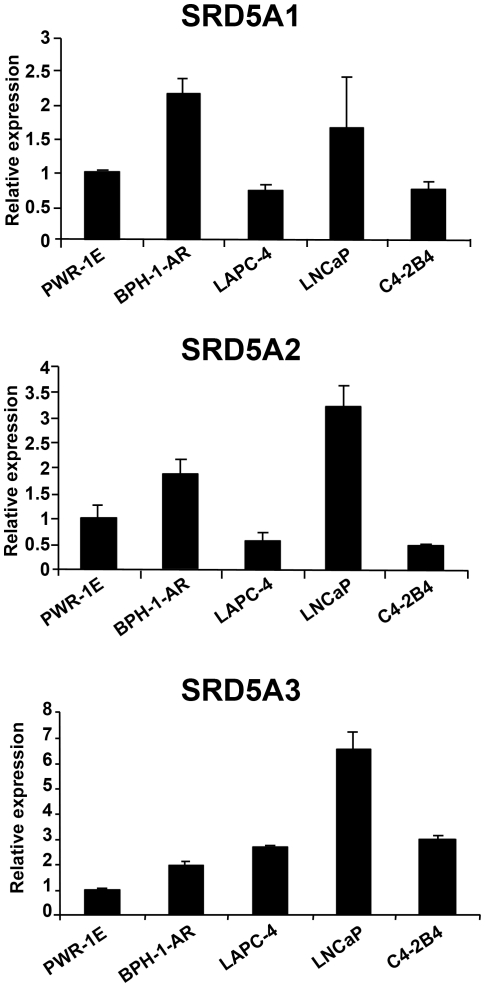
The expression pattern of 5α-reductase in prostate cell lines varies. Graphs depict the relative mRNA levels of the *SRD5A1*, *SRD5A2*, and *SRD5A3* isoenzymes in PWR-1E, BPH-1-AR, LAPC-4, LNCaP, and C4-2B4 cells as determined on qRT-PCR analysis.

To identify good model systems for studying the functions of 5α-reductase isoenzymes, we evaluated the mRNA levels of the 5α-reductase isoenzymes in different prostate cell lines, including PWR-1E immortalized normal prostatic epithelial cells; BPH-1-AR benign prostatic hyperplasia (BPH) cells, which stably express AR; LAPC-4 and LNCaP androgen-sensitive prostate cancer cells; and C4-2B4 androgen-independent cells. PWR1-E, BPH-1-AR and LAPC-4 cells express wild-type AR, whereas LNCaP and C4-2B4 cells express a mutant AR, T877A. (The characteristics of these cell lines, including their source, androgen sensitivity, and AR-expression status, are summarized in Table S1.) As Figure 1 illustrates, the mRNA of all three 5α-reductase isoenzymes was detected on qRT-PCR analysis of each cell line, indicating that all three are expressed in these cell lines. However, the mRNA level of each isoenzyme differed between cell lines, and each isoenzyme had a distinctive expression pattern.

### 5α-Reductase mRNA levels are regulated by androgens in a cell type–specific manner

**Figure 2:**
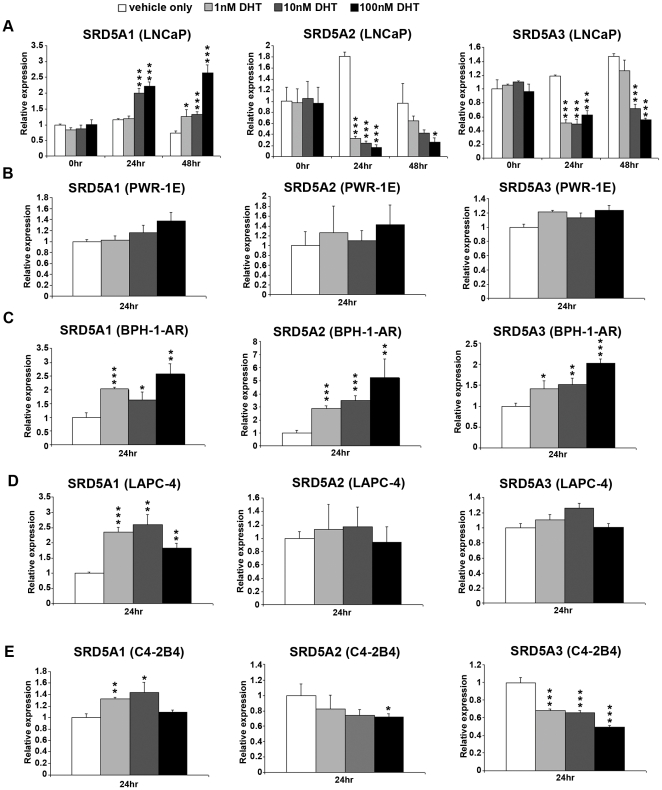
DHT regulates the mRNA level of 5α-reductase differently in different prostate cell lines. A, LNCaP cells were treated with ethanol (vehicle only) or different concentrations of DHT (1 nM, 10 nM, 100 nM) for 24 and 48 hours. PWR-1E (B), BPH-1-AR (C), LAPC-4 (D), and C4-2B4 (E) cells were treated the same way but for only for 24 hours. The mRNA levels of *SRD5A1*, *SRD5A2*, and *SRD5A3* for all cell lines were quantified by qRT-PCR. *p<0.05, **p<0.01, ***p<0.001; 2-sided *t* test.

To test whether the mRNA level of 5α-reductase is regulated by androgens, we treated LNCaP cells with either ethanol (i.e., vehicle only) or with testosterone or DHT at different concentrations (1, 10, and 100 nM). The normal plasma concentration of testosterone in men ranges from 350 to 1050 ng/dL (12.1–36.4 nM) [37], and the castrate level of testosterone is less than 50 ng/dL (1.73 nM) [38]. The plasma concentration of DHT is about 1/10 of the testosterone concentration [39], [40]. Thus, we treated cells with concentrations of androgen that are close to the castrate, physiologic, and superphysiologic levels. Our qRT-PCR analysis showed that DHT treatment resulted in an increased level of *SRD5A1* mRNA, whereas it led to decreased levels of *SRD5A2* and *SRD5A3* mRNA in LNCaP prostate cancer cells (Fig. 2A).

We also tested the effect of DHT on other cell lines. DHT did not notably affect the mRNA level of 5α-reductase in PWR-1E cells (Fig. 2B), whereas in BPH-1-AR cells, DHT increased the mRNA levels of all three isoenzymes (Fig. 2C). In LAPC-4 cells, which express wild-type AR, DHT up-regulated SRD5A1 expression without affecting SRD5A2 and SRD5A3 expression (Fig. 2D). And in the androgen-independent C4-2B4 prostate cancer cells, DHT regulated 5α-reductase expression similarly to its regulation in LNCaP cells, but to a lesser degree (Fig. 2E).

We found it interesting that DHT regulates the mRNA level of *SRD5A3* in a cell type–specific manner, a finding we have not seen reported before. To evaluate whether this happens only in LNCaP or LNCaP-derived cell lines, we similarly treated VCaP cells, which are derived from a xenograft bone metastasis of human prostate cancer. DHT also down-regulated the mRNA level of *SRD5A3* in VCaP cells (Figure S1), indicating that androgen-negative regulation of *SRD5A3* is not specific to LNCaP or LNCaP-derived cell lines.

Testosterone and R1881 had effects similar to those of DHT on the expression of 5α-reductase in all the cell lines tested (Figures S2 and S3). Our data thus demonstrate that androgens regulate the mRNA level of 5α-reductase isoenzymes in a cell type–specific manner.

### Regulation of 5α-reductase mRNA level occurs through transcription

**Figure 3:**
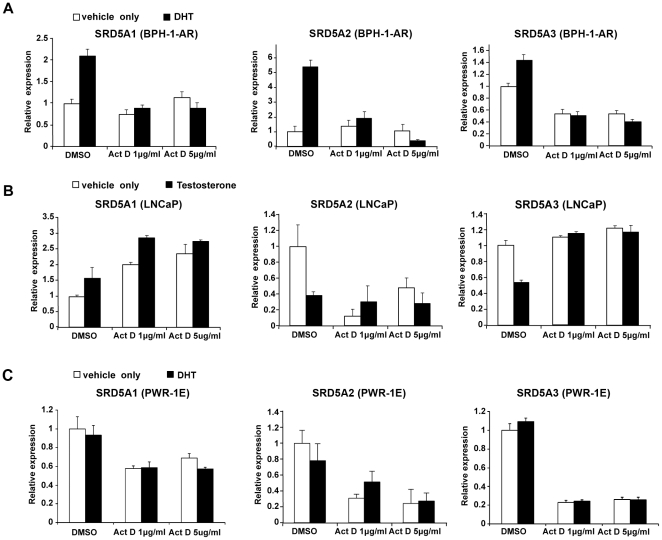
Androgen regulation of 5α-reductase mRNA level is sensitive to actinomycin D treatment. BPH-1-AR (A) and LNCaP (B), and PWR-1E (C) cells were treated for 30 minutes with DMSO (vehicle-only control) or actinomycin D at 1 µg/mL and 5 µg/mL concentrations. Treatment with either ethanol (vehicle only) or 10 nM DHT or testosterone followed. We quantified the mRNA levels of *SRD5A1*, *SRD5A2*, and *SRD5A3* by qRT-PCR.

Androgens could regulate 5α-reductase expression by controlling 5α-reductase transcription or by affecting mRNA stability. To understand the mechanism underlying the regulation of 5α-reductase by androgens, we treated BPH-1-AR cells with actinomycin D, a transcription inhibitor. As shown by the results of qRT-PCR analysis in Figure 3, DHT induced increased expression of all three 5α-reductase isoenzymes relative to that in the ethanol (vehicle) control in BPH-1-AR cells. However, the mRNA level of 5α-reductase did not change with DHT treatment when the cells were also treated with actinomycin D at 1 µg/mL and 5 µg/mL concentrations (Fig. 3A). These results indicate that the up-regulation of 5α-reductase expression by androgens is sensitive to actinomycin D treatment, suggesting that androgens regulate the transcription of 5α-reductase.

Similarly, when LNCaP cells were treated with actinomycin D, testosterone did not significantly increase *SRD5A1* or decrease *SRD5A2* and *SRD5A3* mRNA levels (Fig. 3B). As a negative control, we also treated PWR-1E cells with actinomycin D, followed by DHT treatment (Fig. 3C). In the case of the DMSO-only control, DHT did not affect the mRNA level of *SRD5A1*, *SRD5A2*, or *SRD5A3*. With actinomycin D treatment, DHT did not significantly alter the mRNA level of these genes, either.

### Regulation of 5α-reductase mRNA level by androgens is AR dependent

**Figure 4:**
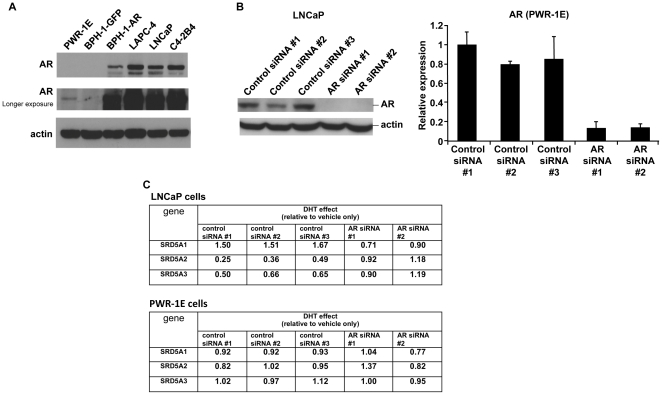
Regulation of 5α-reductase mRNA level by DHT is AR dependent. A, The AR protein level of each prostate cell line was analyzed by Western blotting. BPH-1-GFP cells were used as a negative control for AR expression. B, The AR protein level was analyzed by Western blotting for LNCaP cells (left) with three control and two AR siRNA treatments. The AR mRNA level was analyzed by qRT-PCR for PWR-1E cells (right), also with three control and two AR siRNA treatments. C, LNCaP and PWR-1E cells were treated with control siRNAs and AR siRNAs and then treated with 2 nM DHT. We measured the mRNA levels of *SRD5A1*, *SRD5A2*, and *SRD5A3* by using qRT-PCR and normalized the values to β-actin. The changes in mRNA levels with DHT treatment are shown relative to the levels in cells treated with vehicle only.

By using Western blotting, we verified the expression of AR in each of the studied cell lines by comparison with that of the AR-negative BPH-1-GFP cells (Fig. 4A).

To determine whether AR is required to mediate regulation of 5α-reductase expression by androgens, we performed Western blotting and qRT-PCR analysis after treating LNCaP and PWR-1E cells with AR siRNAs and then with 2 nM DHT. Western blotting and qRT-PCR validated the effective knockdown of AR expression by the AR siRNAs (Fig. 4B). qRT-PCR showed that the control siRNAs did not significantly alter the effect of DHT on 5α-reductase levels in LNCaP cells (Fig. 4C, top). DHT treatment alone resulted in increased *SRD5A1* mRNA but decreased *SRD5A2* and *SRD5A3* mRNA (Fig. 4C, top). In contrast, the 5α-reductase levels in LNCaP cells treated with AR siRNAs did not change in response to treatment with DHT (Fig. 4C, top). When we treated LAPC-4 cells in a similar way, *SRD5A1* mRNA similarly increased with DHT or testosterone treatment alone, but not when cells were also treated with AR siRNA (Figure S4). When we treated PWR-1E cells in the same way, DHT did not affect the mRNA level of 5α-reductase in PWR-1E cells, no matter whether they were treated with control siRNAs or AR siRNAs (Fig. 4C, bottom).

Taken together, these results indicate that the regulation of 5α-reductase by androgens is AR dependent.

### *SRD5A3* promoter contains an nARE

**Figure 5:**
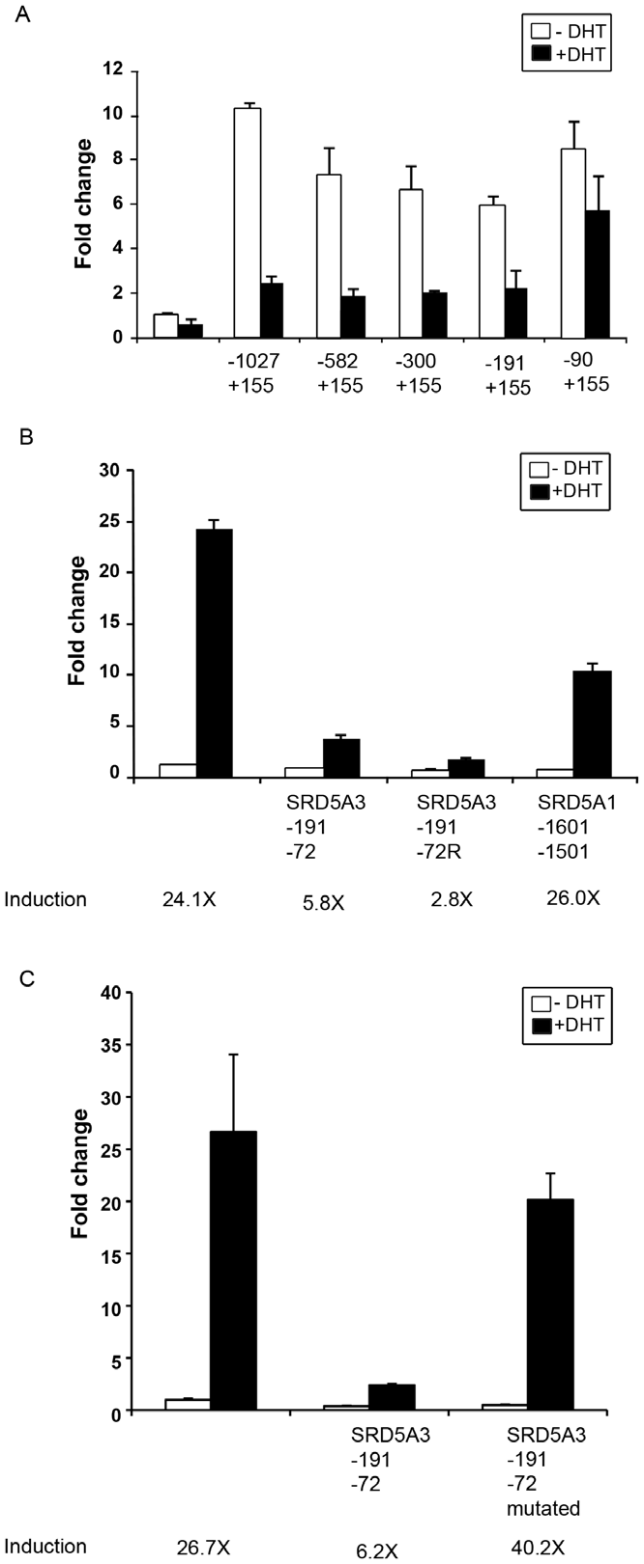
The *SRD5A3* promoter contains a functional nARE. A, Deletion analysis of *SRD5A3* promoter to narrow down the location of the nARE-containing region. LNCaP cells were transfected with luciferase constructs containing a deletion series of *SRD5A3* promoter and then treated with ethanol (vehicle only; −DHT) or 100 nM DHT (+DHT) for 24 hours. Cells were then harvested, and their lysates were used for the luciferase assay. B, *SRD5A3* has an nARE. LNCaP cells were transfected with pGL3–4ARE–E4 constructs with and without insertion of the *SRD5A3* promoter (−191/−72 bp) sequence in both orientations or with insertion of an *SRD5A1* promoter fragment (−1601/−1501 bp). Cells were treated with ethanol (vehicle only) or 100 nM DHT for 24 hours and then harvested for the luciferase assay. C, Mutations in the nARE abolished its suppressive effect. LNCaP cells were transfected with pGL3–4ARE–E4 constructs with and without insertion of *SRD5A3* promoter (−191/−72 bp) or with insertion of the mutated *SRD5A3* promoter (−191/−72 bp) and then subjected to DHT treatment and the luciferase assay.

We showed that androgen regulates the transcription of 5α-reductase and that AR is necessary to mediate this transcriptional regulation. AR directly binds to AREs and regulates the transcription of the AR-targeted genes. Thus, we investigated whether AR can directly regulate the transcription of 5α-reductase. We cloned a promoter region of *SRD5A3* (−1027 to +155 bp) into the pGL2-basic vector. In LNCaP cells, this construct drives luciferase expression and, when they are treated with 100 nM DHT, a 75% reduction of luciferase activity results (Fig. 5A). This is similar to the response of endogenous *SRD5A3* to androgen treatment in LNCaP cells. Thus, an ARE may reside in this 1-kb promoter region of *SRD5A3*.

To identify the putative ARE, we made a series of deletion constructs in the *SRD5A3* promoter region; the constructs retained responsiveness to androgen treatment until the −191/−91 bps were deleted, suggesting that the putative ARE is located in this region (Fig. 5A).

To further evaluate whether this region indeed contains an nARE, we inserted the region of −191/−72 bp between the 4ARE and E4 core promoter in the pGL3–4ARE–E4–luc construct [41], transfected LNCaP cells with it, and then treated the cells with DHT. The pGL3–4ARE–E4–luc construct contains four tandem repeats of the ARE of the *PSA* gene and an E4 core promoter [41]. As shown in Figure 5B, DHT treatment induced about a 24-fold increase in luciferase activity of the pGL3–4ARE–E4–luc construct. When the −191/−72-bp region of the *SRD5A3* promoter was inserted between 4ARE and E4, luciferase activity was increased only 3- to 6-fold by DHT treatment, suggesting that this region contains an nARE. When a 101-bp region derived from the *SRD5A1* promoter (−1601/−1501 bp) was similarly inserted between 4ARE and E4 as a control, DHT treatment still induced a 26-fold increase in luciferase activity. Furthermore, mutations in the −191/−72-bp region of *SRD5A3* abolished its repressive ability (Fig. 5C). Together, these data suggest that *SRD5A3* has a functional nARE in its promoter.

### AR is recruited to the nARE-containing region of *SRD5A3*

**Figure 6:**
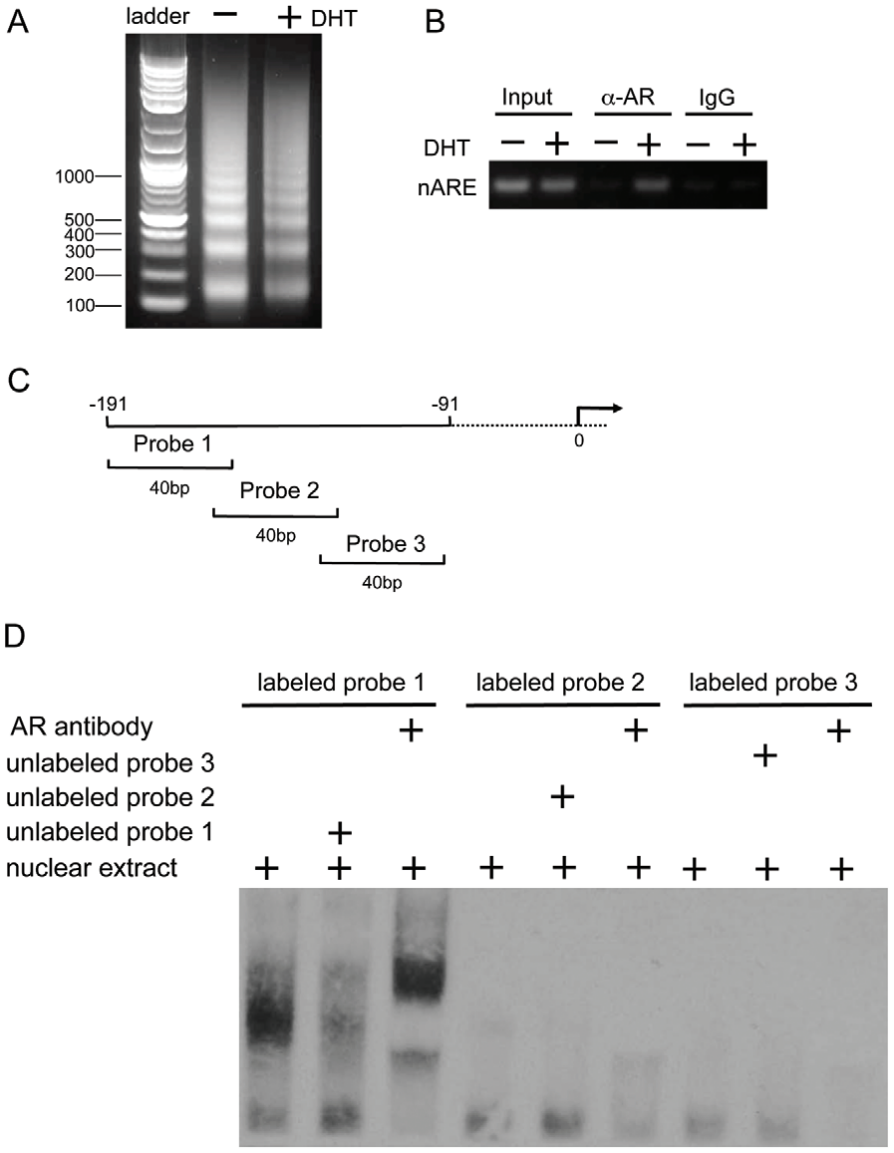
AR directly interacts with the nARE of the *SRD5A3* gene. A, Agarose gel electrophoresis of the digested chromatin of LNCaP cells grown in the absence and presence of 100 nM DHT. DNA fragments generally range between 150 and 900 bp. B, Digested chromatin of LNCaP cells grown in the absence and presence of 100 nM DHT was used in the immunoprecipitation experiment with anti-AR antibody or normal mouse immunoglobulin G. Afterward, the protein–DNA crosslink was reversed, and purified DNA was used in the PCR reactions with primers flanking the nARE region. C, Three oligo probes were designed to cover the −191/−91 bp region in the promoter of *SRD5A3*. D, AR binds to the nARE of *SRD5A3*. The three (biotin-labeled) oligo probes were incubated with LNCaP cell nuclear extract, with LNCaP cell nuclear extract plus unlabeled oligo probes, or with LNCaP cell nuclear extract plus AR-specific antibody.

To investigate whether AR is recruited to the promoter of *SRD5A3 in vivo*, we performed ChIP using genomic DNA fragments from LNCaP cells (150–900 bp; Fig. 6A) with primers specifically targeting the nARE region of *SRD5A3*. As shown on PCR, AR was enriched at the nARE region when cells grew in the presence of DHT (Fig. 6B). Normal mouse immunoglobulin G (as a negative control) was also used for immunoprecipitation with LNCaP genomic DNA; the immunoglobulin G did not pull down any AR-associated DNA sequences of the *SRD5A3* promoter (Fig. 6B). These results suggest that AR is recruited to the nARE region in the promoter of *SRD5A3*.

To determine whether AR can directly interact with the nARE of *SRD5A3*, we carried out EMSA with three oligo probes, each of 40 bp, to cover the −191/−91-bp region of the *SRD5A3* promoter (Fig. 6C). Only probe 1 showed a mobility shift (Fig. 6D). To confirm that this mobility shift is specific to AR, we added anti-AR antibodies to the reactions; probe 1 showed a further mobility shift (Fig. 6D). Although unlabeled wild-type probe 1 was able to compete with labeled probe 1 for AR binding in EMSA, it lost that ability when we made mutations in the probe 1 sequence (Figure S5).

These results demonstrated that probe 1 contains the nARE and that AR directly binds to this region *in vitro*.

For *SRD5A1* and *SRD5A2*, we also conducted promoter analysis and ChIP, but we did not detect an ARE region or direct AR binding to their proximal promoter regions. The regulational mechanism for their expression is still under investigation.

### *SRD5A3* mRNA increases in the AR-negative SCPC xenograft model

**Figure 7:**
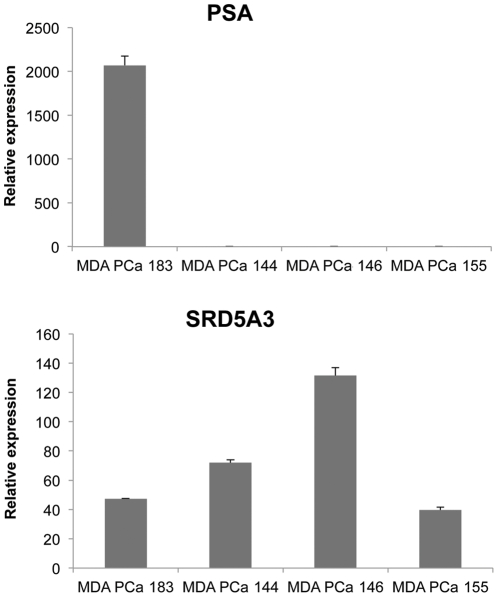
The *SRD5*A3 mRNA level is lower in androgen-dependent xenografts than it is in androgen-independent AR-negative SCPC xenografts. *PSA* (top) and *SRD5A3* (bottom) mRNA levels were analyzed by qRT-PCR for the androgen-dependent xenograft MDA PCa 183 and the androgen-independent AR-negative SCPC xenografts MDA PCa 144, MDA PCa 146, and MDA PCa 155.

On observing the androgen-negative regulation of *SRD5A3* in the tested cell lines, we were interested in investigating whether this AR-dependent regulation also occurs *in vivo*. Therefore, we examined the mRNA level of *SRD5A3* in different xenograft models, including MDA PCa 183, an AR-expressing androgen-dependent prostate cancer xenograft, and three AR-negative androgen-independent SCPC xenografts, MDA PCa 144, MDA PCa 146, and MDA PCa 155. On qRT-PCR, we observed a remarkably higher mRNA level of *PSA* in the MDA PCa 183 xenograft than in the others (Fig. 7, top), which is consistent with the AR status of these cell lines. In contrast, the mRNA level of *SRD5A3* in the AR-positive xenograft MDA PCa 183 was much lower than that in the AR-negative SCPC xenografts, except for MDA PCa 155 (Fig. 7, bottom), suggesting a loss of AR-negative regulation in the MDA PCa 144 and MDA PCa 146 xenografts. This result is consistent with the mechanism underlying the androgen-negative regulation of *SRD5A3* expression that we found in the prostate cancer cell lines.

## Discussion

The results of this study show that androgens regulate the expression of 5α-reductase isoenzymes in a cell type–specific manner. They also show that androgens regulate the transcription of 5α-reductase isoenzymes and that such regulation is mediated by the AR. Furthermore, to our knowledge, this is the first publication of evidence that *SRD5A3* has at least one nARE and that AR directly binds to this nARE region, demonstrating that *SRD5A3* is under transcription inhibition by AR in prostate cancer cells. Altogether, our results elucidate a mechanism by which androgens regulate the expression level of 5α-reductase isoenzymes; this may have clinical implications, by revealing a mechanistic basis for the response and/or resistance to 5α-reductase inhibitors.

In this study, the mRNA level of 5α-reductase did not fully correlate with previously reported immunostaining results in human tissues [8], but it is possible that the mRNA level during different stages of prostate cancer development and progression does not fully correlate with the protein levels as measured by immunostaining. Luo and colleagues [7] detected no significant difference in the *SRD5A1* mRNA level between tumor samples and BPH or normal samples, although Thomas et al. [8] found greater SRD5A1 immunostaining in tumor samples than they found in BPH samples. It is also possible that the mRNA level of 5α-reductase in this study was modified because we used immortalized cell lines. Additionally, it remains controversial how the 5α-reductase level changes with prostate cancer development and progression, as shown in a recent study by Wako et al. [42]: using semiquantitative immunohistochemical analysis, they found no significant change in SRD5A1 and SRD5A2 levels between localized prostate cancer samples and normal prostatic tissues.

This study evaluated multiple cell lines that may represent the processes of prostate cancer initiation and progression. Although these are immortalized cell lines, the results still shed light on how androgen may modulate the mRNA level of 5α-reductase during different stages of prostate cancer pathogenesis and progression. In PWR-1E cells, for example, androgens did not have a significant effect on 5α-reductase expression. However, in BPH-1-AR cells, which represent the transition between normal and cancerous prostate cells,testosterone could up-regulate the mRNA levels of all three 5α-reductase isoenzymes, which would result in high concentrations of DHT in these cells and further increase the expression of these isoenzymes. Such a positive-feedback loop is most likely to lead to constant activation of the AR, which at least partially explains the epidemiologic observation that a high concentration of testosterone level correlates with a high incidence of prostate tumor [43]. In our experiments, the LAPC-4, LNCaP, and C4-2B4 prostate cancer cell lines responded differently to androgen treatment. They also have different androgen sensitivity and AR status, which is an important implication that blocking DHT production with either 5α-reductase inhibitor may not be the gold-standard prevention strategy in every man because the androgen sensitivity, AR status and other factors of each particular prostate cancer may influence expression of 5α-reductase isoenzymes in reponses to blocking of DHT production.

Several genes, including *PSA*, *TGF-β1*, and *maspin*, reportedly contain an nARE in their *cis*-regulatory elements [41], [44], [45]. The nARE in the *PSA* gene overlaps with the NF-κB binding site, and AR competes with NF-κB binding, resulting in the negative regulation of *PSA* [44]. A 250-bp nARE region has also been mapped to the promoter of *TGF-β1*, although no direct binding of AR to this region has been detected [41]. Further, the AR binds to the nARE of the *maspin* promoter [45]. However, the nARE in the promoter of *SRD5A3* has no notable similarity to that in *PSA*, *TGF-β1*, or *maspin*. Therefore, we believe that we have found a novel nARE and have demonstrated the direct binding of the AR to this region.

The *SRD5A3* mRNA level was not consistently significantly affected by androgens in PWR-1E cells, was up-regulated in BPH-1-AR cells, but was down-regulated in LNCaP cells. These results imply a transition in the regulation of *SRD5A3* expression with prostate cancer pathogenesis and progression—a loss of positive regulation and gain of negative regulation—which is consistent with the report that a lower testosterone level was found among prostate cancer patients with higher Gleason scores than that in those with lower Gleason scores [46]. It is also consistent with the observation that *SRD5A3* is overexpressed in hormone-refractory prostate cancers in which the androgen level is low [10], [11]. Our identification of an nARE in the promoter of *SRD5A3* allows us to further investigate the transition from positive to negative regulation of *SRD5A3* expression that occurs with prostate cancer progression over time.

The results of our evaluation of xenograft models provide additional supporting evidence of the androgen-negative regulation of *SRD5A3*. Because we did not use castrate xenograft models and thus could not directly compare the effect of androgen withdrawal on the AR-positive and -negative cells *in vivo*, we did observe *SRD5A3* overexpression in the AR-negative prostate carcinoma xenografts MDA PCa 144 and MDA PCa 146, which is consistent with results from our cell line study and previously reported work [10], [11]. Although morphologically similar, the original donor tumor and the MDA PCa 155 subline xenograft have slight differences from the MDA PCa 144 and MDA PCa 146 xenografts in terms of molecular characterization (person al communication with Dr. Sankar N. Maity; a separate manuscript under review elsewhere). It is possible that such differences have an effect on the expression of *SRD5A3*, resulting in the relatively low level of SRD5A3 in MDA PCa 155.

In this study, we did not detect an ARE in the proximal promoter of *SRD5A1* and *SRD5A2*, either by promoter analysis or ChIP assay. It is possible that AR indirectly regulates the transcription of *SRD5A1* and *SRD5A2*, which is mediated by AR downstream transcription factors. It is also possible that AR can directly regulate the transcription of *SRD5A1* and *SRD5A2* but with AREs located in the distal promoter, enhancer, or introns of the genes, which were not uncovered by our study.

Squelching may be another mechanistic possibility for AR-mediated transcription repression. AR has been reported to interact with the general transcription factor TFIIF [47], and AR can compete with T-cell factor (TCF) for binding with β-catenin, thus leading to the suppression of β-catenin/TCF-related transcription [48]. For negative regulation of *SRD5A3* gene expression, we cannot rule out the possibility that AR competes for interaction with general transcription factors, thus interfering with the transcription of *SRD5A3*. However, given the direct binding of AR to the promoter of *SRD5A3* as suggested by our ChIP and EMSA experiments, we believe that such a regulatory mechanism is unlikely.

*SRD5A3* is reportedly overexpressed in hormone-refractory samples from patients [10], [11]. These reports suggest potential stage-dependent expression of 5α-reductase type in the prostate from *SRD5A2* to *SRD5A1* and *SRD5A3*, which is consistent with the fact that the use of 5α-reductase inhibitors is more relevant in early than in more advanced prostate cancers. It remains controversial whether SRD5A3 enzyme activity can be inhibited by finasteride or dutasteride. Although Yamana et al. reported that finasteride and dutasteride can block the activity of SRD5A3 [49], Titus and Mohler [11] stated in a book chapter that the 5α-reductase inhibitor dutasteride does not inhibit the activity of SRD5A3. The presence and expression level of *SRD5A3*, which is dependent on the presence of androgens (i.e., repression of *SRD5A3* by androgens), could be an important contributor to resistance to therapy with a 5α-reductase inhibitor. Therefore, the role of *SRD5A3* in both prostate cancer progression and prevention is worth further investigation.

The differing expression of 5α-reductase isoenzymes may also contribute to response or resistance to 5α-reductase inhibitors. Because AR regulates 5α-reductase expression in a cell type–dependent manner, we would not expect to prevent prostate cancer in all men by administering finasteride (a specific SRD5A2 inhibitor) or dutasteride (an inhibitor of both SRD5A1 and SRD5A2); this concurs with the results of the PCPT and REDUCE trials. Additionally, whether the androgen regulation of 5α-reductase isoenzymes is testosterone- or DHT-driven needs further investigation. It also remains to be seen whether 5α-reductases are regulated by AR in a ligand-independent manner. This points to the fact that we may need combination therapy, possibly inhibiting 5α-reductase isoenzymes, the AR, and other factors involved in ligand-independent AR activation for efficient prevention of prostate cancer in all men.

## Supporting Information

Figure S1 **DHT regulates the mRNA level of 5α-reductase in VCaP cells.** In this experiment, VCaP cells were treated with ethanol (vehicle only) or with 1 nM, 10 nM, or 100 nM DHT for 24 hours. We quantified the mRNA levels of *SRD5A1*, *SRD5A2*, and *SRD5A3* by using qRT-PCR. *p<0.05, ** p<0.01, *** p<0.001; 2-sided *t* test.
(TIF)

Figure S2 **Testosterone regulates the mRNA level of 5α-reductase differently in different prostate cell lines.** LNCaP (A), PWR-1E (B), BPH-1-AR (C), LAPC-4 (D), and C4-2B4 (E) cells were treated with ethanol (vehicle only) or with 1 nM, 10 nM, or 100 nM testosterone for 24 hours. The mRNA levels of *SRD5A1*, *SRD5A2*, and *SRD5A3* for all cells were quantified by using qRT-PCR. *p<0.05, **p<0.01, ***p<0.001; 2-sided *t* test.
(TIF)

Figure S3 **The synthetic androgen R1881 regulates the mRNA level of 5α-reductase differently in different prostate cell lines.** LNCaP (A), PWR-1E (B), BPH-1-AR (C), LAPC-4 (D), and C4-2B4 (E) cells were treated with ethanol (vehicle only) or 1 nM, 10 nM, or 100 nM R1881for 24 hours. The mRNA levels of *SRD5A1*, *SRD5A2*, and *SRD5A3* for all cells were quantified by using qRT-PCR. *p<0.05, **p<0.01, ***p<0.001; 2-sided *t* test.
(TIF)

Figure S4 **Regulation of 5α-reductase mRNA level by DHT is AR dependent in LAPC-4 cells.** A, The AR protein level was analyzed by Western blotting with no siRNA, control siRNA, and four AR siRNA treatments. AR siRNA#1 and AR siRNA#2 had a stronger knockdown effect than the other two AR siRNAs did. B, LAPC-4 cells were treated with no siRNA, control siRNA or with AR siRNAs (siRNAs #1 and #2), followed by treatment with 2 nM DHT. The mRNA level of *SRD5A1* was measured by using qRT-PCR and normalized to β-actin. The changes in mRNA levels resulting from DHT treatment are shown relative to the levels in cells treated with vehicle only.
(TIF)

Figure S5 **Mutations in the nARE of** ***SRD5A3*** **impair its binding with the AR.** Mutations were made in the sequence of *SRD5A3* oligo probe 1. In EMSA, biotin-labeled oligo probe 1 was incubated alone (lane 1), with LNCaP cell nuclear extract (lane 2), with LNCaP cell nuclear extract and unlabeled oligo probe 1 (lane 3), or with LNCaP cell nuclear extract plus unlabeled mutated oligo probe 1 (lane 4).
(TIF)

Table S1 **Characteristics of the cell lines.**
(DOC)

## References

[1] Fitzpatrick JM, Schulman C, Zlotta AR, Schroder FH (2009). Prostate cancer: a serious disease suitable for prevention.. BJU Int.

[2] Pienta KJ, Bradley D (2006). Mechanisms underlying the development of androgen-independent prostate cancer.. Clin Cancer Res.

[3] Scher HI, Sawyers CL (2005). Biology of progressive, castration-resistant prostate cancer: directed therapies targeting the androgen-receptor signaling axis.. J Clin Oncol.

[4] Zhu YS, Imperato-McGinley JL (2009). 5α-Reductase isozymes and androgen actions in the prostate.. Ann N Y Acad Sci.

[5] Wilson EM, French FS (1976). Binding properties of androgen receptors. Evidence for identical receptors in rat testis, epididymis, and prostate.. J Biol Chem.

[6] Wilbert DM, Griffin JE, Wilson JD (1983). Characterization of the cytosol androgen receptor of the human prostate.. J Clin Endocrinol Metab.

[7] Luo J, Dunn TA, Ewing CM, Walsh PC, Isaacs WB (2003). Decreased gene expression of steroid 5 alpha-reductase 2 in human prostate cancer: implications for finasteride therapy of prostate carcinoma.. Prostate.

[8] Thomas LN, Lazier CB, Gupta R, Norman RW, Troyer DA (2005). Differential alterations in 5α-reductase type 1 and type 2 levels during development and progression of prostate cancer.. Prostate.

[9] Bjelfman C, Soderstrom TG, Brekkan E, Norlen BJ, Egevad L (1997). Differential gene expression of steroid 5 alpha-reductase 2 in core needle biopsies from malignant and benign prostatic tissue.. J Clin Endocrinol Metab.

[10] Uemura M, Tamura K, Chung S, Honma S, Okuyama A (2008). Novel 5α-steroid reductase (SRD5A3, type-3) is overexpressed in hormone-refractory prostate cancer.. Cancer Sci.

[11] Titus MA, Mohler JL (2009). 5α-Reductase Isozymes in Castration-Recurrent Prostate Cancer..

[12] Godoy A, Kawinski E, Li Y, Oka D, Alexiev B (2011). 5alpha-reductase type 3 expression in human benign and malignant tissues: a comparative analysis during prostate cancer progression.. Prostate.

[13] Cantagrel V, Lefeber DJ, Ng BG, Guan Z, Silhavy JL (2010). SRD5A3 is required for converting polyprenol to dolichol and is mutated in a congenital glycosylation disorder.. Cell.

[14] Morava E, Wevers RA, Cantagrel V, Hoefsloot LH, Al-Gazali L (2010). A novel cerebello-ocular syndrome with abnormal glycosylation due to abnormalities in dolichol metabolism.. Brain.

[15] Kahrizi K, Hu CH, Garshasbi M, Abedini SS, Ghadami S (2011). Next generation sequencing in a family with autosomal recessive Kahrizi syndrome (OMIM 612713) reveals a homozygous frameshift mutation in SRD5A3.. Eur J Hum Genet.

[16] Stoner E (1990). The clinical development of a 5α-reductase inhibitor, finasteride.. J Steroid Biochem Mol Biol.

[17] Tian G, Mook RA, Moss ML, Frye SV (1995). Mechanism of time-dependent inhibition of 5 alpha-reductases by delta 1–4-azasteroids: toward perfection of rates of time-dependent inhibition by using ligand-binding energies.. Biochemistry.

[18] Thompson IM, Goodman PJ, Tangen CM, Lucia MS, Miller GJ (2003). The influence of finasteride on the development of prostate cancer.. N Engl J Med.

[19] Andriole G, Bostwick D, Brawley O, Gomella L, Marberger M (2004). Chemoprevention of prostate cancer in men at high risk: rationale and design of the Reduction by Dutasteride of Prostate Cancer Events (REDUCE) trial.. J Urol.

[20] Andriole GL, Bostwick DG, Brawley OW, Gomella LG, Marberger M (2010). Effect of dutasteride on the risk of prostate cancer.. N Engl J Med.

[21] Xu Y, Dalrymple SL, Becker RE, Denmeade SR, Isaacs JT (2006). Pharmacologic basis for the enhanced efficacy of dutasteride against prostatic cancers.. Clin Cancer Res.

[22] Makridakis N, Reichardt JKV (2005). Pharmacogenetic analysis of human steroid 5α reductase type II: comparison of finasteride and dutasteride.. J Mol Endocrinol.

[23] Makridakis NM, di Salle E, Reichardt JKV (2000). Biochemical and pharmacogenetic dissection of human steroid 5α-reductase type II.. Pharmacogenetics.

[24] George FW (1997). Androgen metabolism in the prostate of the finasteride-treated, adult rat: a possible explanation for the differential action of testosterone and 5 alpha-dihydrotestosterone during development of the male urogenital tract.. Endocrinology.

[25] Steiner JF (1996). Clinical pharmacokinetics and pharmacodynamics of finasteride.. Clin Pharmacokinet.

[26] Rittmaster R, Hahn RG, Ray P, Shannon JB, Wurzel R (2008). Effect of dutasteride on intraprostatic androgen levels in men with benign prostatic hyperplasia or prostate cancer.. Urology.

[27] George FW, Russell DW, Wilson JD (1991). Feed-forward control of prostate growth: dihydrotestosterone induces expression of its own biosynthetic enzyme, steroid 5α-reductase.. Proc Natl Acad Sci U S A.

[28] Pratis K, O'Donnell L, Ooi GT, Stanton PG, McLachlan RI (2003). Differential regulation of rat testicular 5α-reductase type 1 and 2 isoforms by testosterone and FSH.. J Endocrinol.

[29] Silver RI, Wiley EL, Davis DL, Thigpen AE, Russell DW (1994). Expression and regulation of steroid 5 alpha-reductase 2 in prostate disease.. J Urol.

[30] Zhou Z, Speiser PW (1999). Regulation of HSD17B1 and SRD5A1 in lymphocytes.. Mol Genet Metab.

[31] Melcangi RC, Poletti A, Cavarretta I, Celotti F, Colciago A (1998). The 5alpha-reductase in the central nervous system: expression and modes of control.. J Steroid Biochem Mol Biol.

[32] Torres JM, Ortega E (2003). Precise quantitation of 5α-reductase type 1 mRNA by RT-PCR in rat liver and its positive regulation by testosterone and dihydrotestosterone.. Biochem Biophys Res Commun.

[33] Torres JM, Ortega E (2003). Differential regulation of steroid 5α-reductase isozymes expression by androgens in the adult rat brain.. Faseb J.

[34] El-Awady MK, El-Garf W, El-Houssieny L (2004). Steroid 5α reductase mRNA type I is differentially regulated by androgens and glucocorticoids in the rat liver.. Endocr J.

[35] Romano P, Manniello A, Aresu O, Armento M, Cesaro M (2009). Cell Line Data Base: structure and recent improvements towards molecular authentication of human cell lines.. Nucleic Acids Res.

[36] Aparicio A, Tzelepi V, Araujo JC, Guo CC, Liang S (2011). Neuroendocrine prostate cancer xenografts with large-cell and small-cell features derived from a single patient's tumor: morphological, immunohistochemical, and gene expression profiles.. Prostate.

[37] Dai WS, Kuller LH, LaPorte RE, Gutai JP, Falvo-Gerard L (1981). The epidemiology of plasma testosterone levels in middle-aged men.. Am J Epidemiol.

[38] Reddy GK (2004). Abarelix (Plenaxis): a gonadotropin-releasing hormone antagonist for medical castration in patients with advanced prostate cancer.. Clin Prostate Cancer.

[39] Wilson JD (1996). Role of dihydrotestosterone in androgen action.. Prostate.

[40] Noss KR, Wolfe SA, Grimes SR (2002). Upregulation of prostate specific membrane antigen/folate hydrolase transcription by an enhancer.. Gene.

[41] Qi W, Gao S, Wang Z (2008). Transcriptional regulation of the TGF-β1 promoter by androgen receptor.. Biochem J.

[42] Wako K, Kawasaki T, Yamana K, Suzuki K, Jiang S (2008). Expression of androgen receptor through androgen-converting enzymes is associated with biological aggressiveness in prostate cancer.. J Clin Pathol.

[43] Parsons JK, Carter HB, Platz EA, Wright EJ, Landis P (2005). Serum testosterone and the risk of prostate cancer: potential implications for testosterone therapy.. Cancer Epidemiol Biomarkers Prev.

[44] Cinar B, Yeung F, Konaka H, Mayo MW, Freeman MR (2004). Identification of a negative regulatory cis-element in the enhancer core region of the prostate-specific antigen promoter: implications for intersection of androgen receptor and nuclear factor-κB signalling in prostate cancer cells.. Biochem J.

[45] Zhang M, Magit D, Sager R (1997). Expression of maspin in prostate cells is regulated by a positive Ets element and a negative hormonal responsive element site recognized by androgen receptor.. Proc Natl Acad Sci U S A.

[46] Schatzl G, Madersbacher S, Thurridl T, Waldmuller J, Kramer G (2001). High-grade prostate cancer is associated with low serum testosterone levels.. Prostate.

[47] Reid J, Murray I, Watt K, Betney R, McEwan IJ (2002). The androgen receptor interacts with multiple regions of the large subunit of general transcription factor TFIIF.. J Biol Chem.

[48] Chesire DR, Isaacs WB (2002). Ligand-dependent inhibition of beta-catenin/TCF signaling by androgen receptor.. Oncogene.

[49] Yamana K, Labrie F, Luu-The V (2010). Human type 3 5αreductase is expressed in peripheral tissues at higher levels than type 1 and type 2 and its activity is potently inhibited by finasteride and dutasteride.. Horm Mol Biol Clin Invest.

