# Supplementary figures and images for: Androgen Regulation of 5α-Reductase Isoenzymes in Prostate Cancer: Implications for Prostate Cancer Prevention
Source: PLoS One. 2011 Dec 14;6(12):e28840. doi: 10.1371/journal.pone.0028840 (PMC3237548; doi:10.1371/journal.pone.0028840)

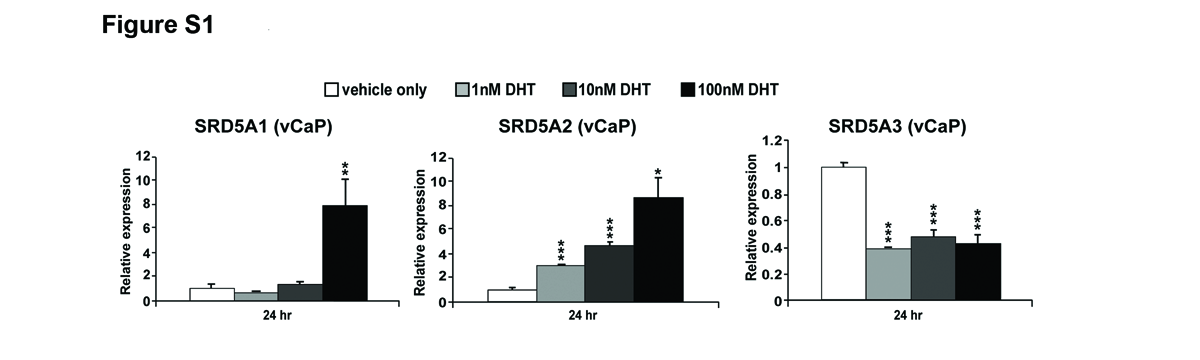

Supplement: Figure S1 — DHT regulates the mRNA level of 5α-reductase in VCaP cells. In this experiment, VCaP cells were treated with ethanol (vehicle only) or with 1 nM, 10 nM, or 100 nM DHT for 24 hours. We quantified the mRNA levels of SRD5A1, SRD5A2, and SRD5A3 by using qRT-PCR. *p<0.05, ** p<0.01, *** p<0.001; 2-sided t test. (TIF) [file pone.0028840.s001.tif]

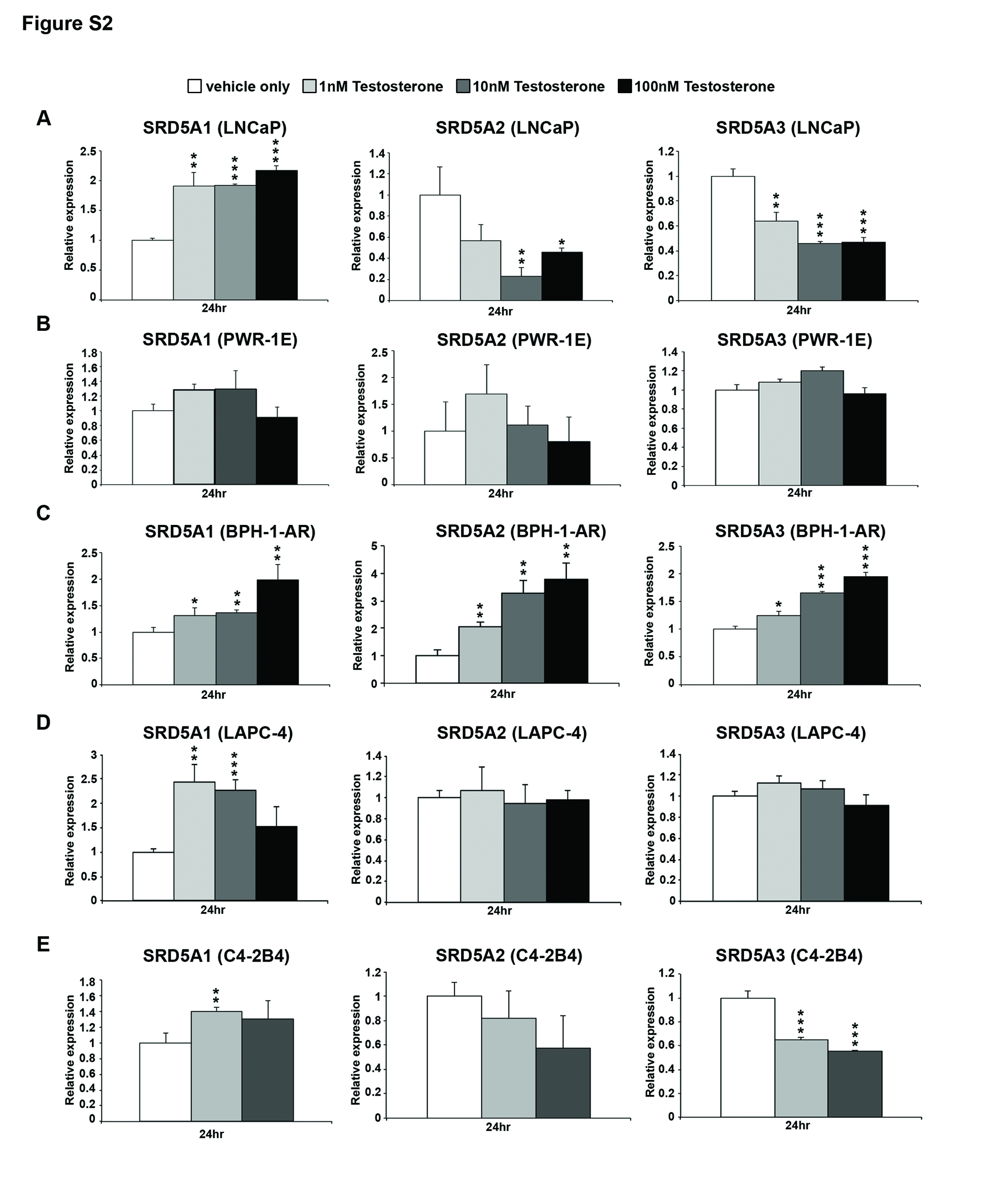

Supplement: Figure S2 — Testosterone regulates the mRNA level of 5α-reductase differently in different prostate cell lines. LNCaP (A), PWR-1E (B), BPH-1-AR (C), LAPC-4 (D), and C4-2B4 (E) cells were treated with ethanol (vehicle only) or with 1 nM, 10 nM, or 100 nM testosterone for 24 hours. The mRNA levels of SRD5A1, SRD5A2, and SRD5A3 for all cells were quantified by using qRT-PCR. *p<0.05, **p<0.01, ***p<0.001; 2-sided t test. (TIF) [file pone.0028840.s002.tif]

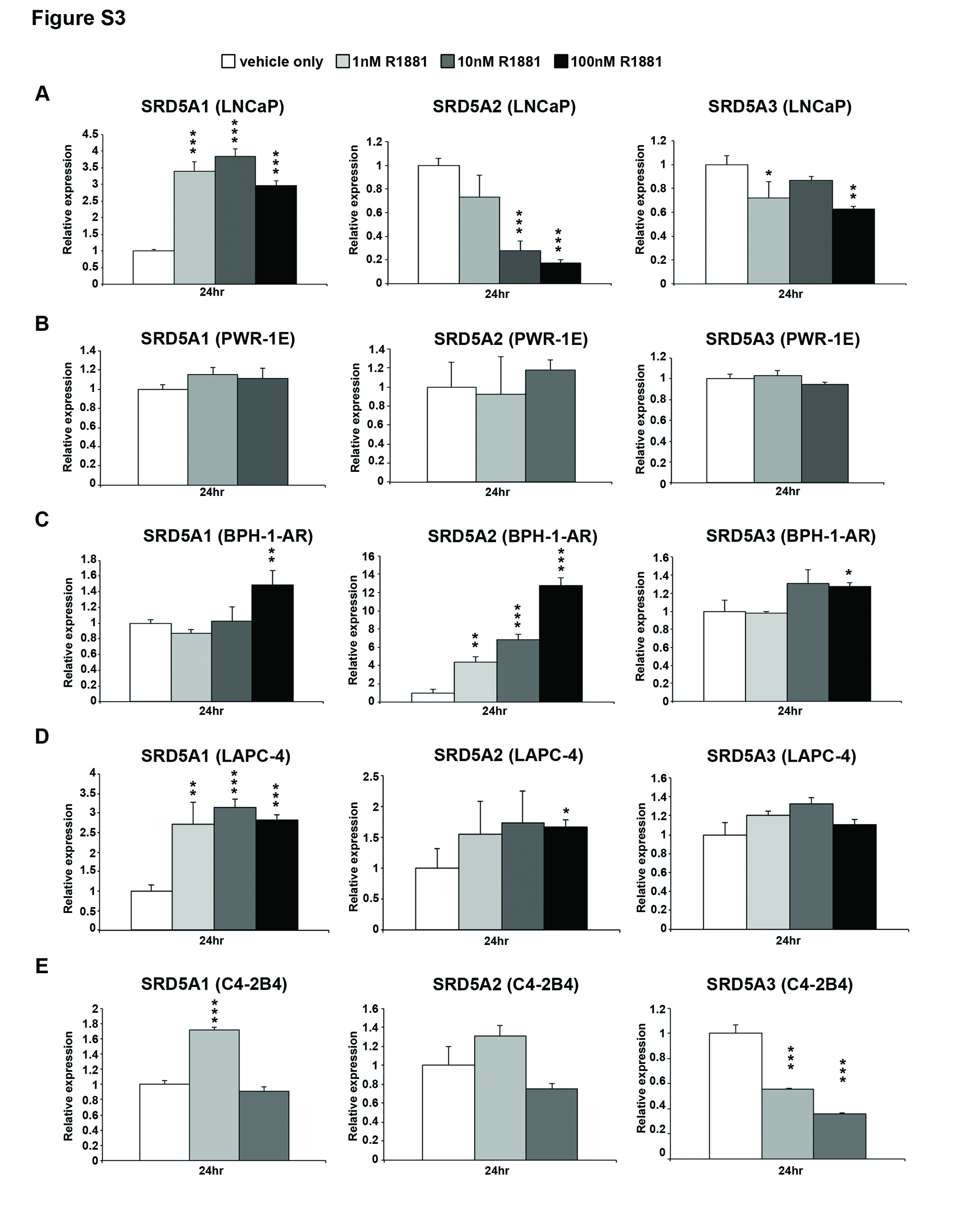

Supplement: Figure S3 — The synthetic androgen R1881 regulates the mRNA level of 5α-reductase differently in different prostate cell lines. LNCaP (A), PWR-1E (B), BPH-1-AR (C), LAPC-4 (D), and C4-2B4 (E) cells were treated with ethanol (vehicle only) or 1 nM, 10 nM, or 100 nM R1881for 24 hours. The mRNA levels of SRD5A1, SRD5A2, and SRD5A3 for all cells were quantified by using qRT-PCR. *p<0.05, **p<0.01, ***p<0.001; 2-sided t test. (TIF) [file pone.0028840.s003.tif]

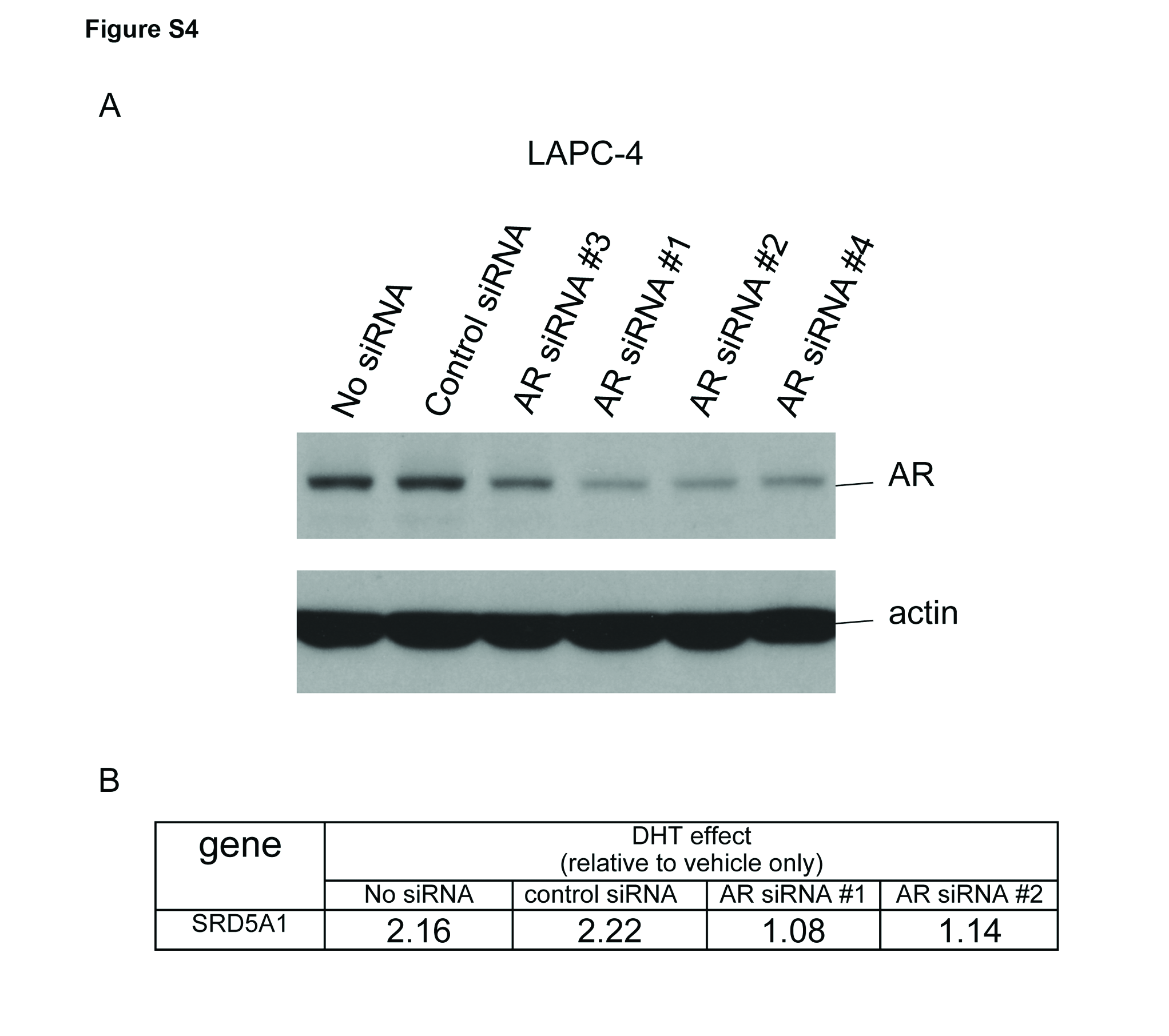

Supplement: Figure S4 — Regulation of 5α-reductase mRNA level by DHT is AR dependent in LAPC-4 cells. A, The AR protein level was analyzed by Western blotting with no siRNA, control siRNA, and four AR siRNA treatments. AR siRNA#1 and AR siRNA#2 had a stronger knockdown effect than the other two AR siRNAs did. B, LAPC-4 cells were treated with no siRNA, control siRNA or with AR siRNAs (siRNAs #1 and #2), followed by treatment with 2 nM DHT. The mRNA level of SRD5A1 was measured by using qRT-PCR and normalized to β-actin. The changes in mRNA levels resulting from DHT treatment are shown relative to the levels in cells treated with vehicle only. (TIF) [file pone.0028840.s004.tif]

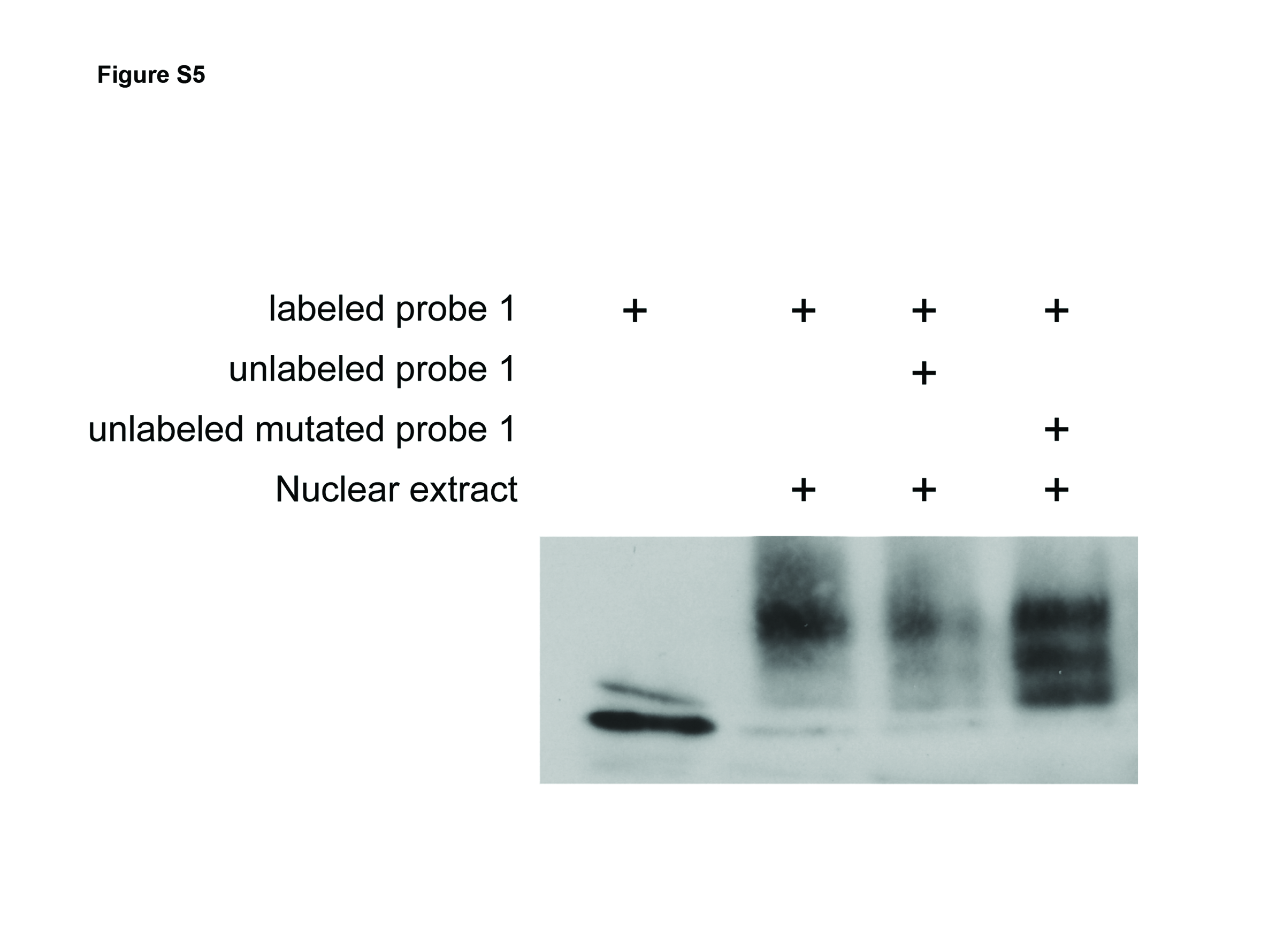

Supplement: Figure S5 — Mutations in the nARE of SRD5A3 impair its binding with the AR. Mutations were made in the sequence of SRD5A3 oligo probe 1. In EMSA, biotin-labeled oligo probe 1 was incubated alone (lane 1), with LNCaP cell nuclear extract (lane 2), with LNCaP cell nuclear extract and unlabeled oligo probe 1 (lane 3), or with LNCaP cell nuclear extract plus unlabeled mutated oligo probe 1 (lane 4). (TIF) [file pone.0028840.s005.tif]
