# Supplementary material for: Androgen Regulation of 5α-Reductase Isoenzymes in Prostate Cancer: Implications for Prostate Cancer Prevention
Source: PLoS One. 2011 Dec 14;6(12):e28840. doi: 10.1371/journal.pone.0028840 (PMC3237548; doi:10.1371/journal.pone.0028840)
Supplement: Table S1 — Characteristics of the cell lines. (DOC) [file pone.0028840.s006.doc]

Table S1. Characteristics of the cell lines

| **Cell Line** | **Source** | **Androgen Sensitivity** | **AR Expression** |
| --- | --- | --- | --- |
| PWR-1E | Immortalized adult human normal prostate epithelial cells | AS | WT |
| BPH-1-AR | Benign prostatic hyperplasia cells with stable expression of AR | AS | WT |
| LAPC-4 | Xenograft lymph node metastasis | AS | WT |
| LNCaP | Lymph node metastasis | AS | Mutant (T877A) |
| C4-2B4  VCaP | Bone metastasis of castrated male mouse injected with passage of LNCaP  vertebral bone metastasis | AI  AS | Mutant (T877A)  WT |

AR, androgen receptor; AS, androgen sensitive; WT, wild type; AI, androgen independent.
